# The negative linear relationship between oxidative balance scores and constipation: a cross-sectional study from NHANES 2005–2010

**DOI:** 10.3389/fmed.2024.1471343

**Published:** 2024-11-15

**Authors:** Liqian Xuan, Yang Chen, Chang Liu, Yahui Dai

**Affiliations:** ^1^Digestive Endoscopy Center, Department of Gastroenterology, Shuguang Hospital Affiliated to Shanghai University of Traditional Chinese Medicine, Shanghai, China; ^2^Department of Emergency, Yueyang Hospital of Integrated Traditional Chinese and Western Medicine, Shanghai University of Traditional Chinese Medicine, Shanghai, China; ^3^Department of Gastroenterology, Shuguang Hospital Affiliated to Shanghai University of Traditional Chinese Medicine, Shanghai, China; ^4^Department of Orthopedics, Songjiang Hospital Affiliated to Shanghai Jiao Tong University School of Medicine, Shanghai, China

**Keywords:** oxidation balance score, constipation, cross-sectional analysis, National Health and Nutrition Examination Survey, digestive health

## Abstract

**Background:**

This study aimed to investigate the relationship between oxidation balance score (OBS) and constipation.

**Methods:**

All data was collected from the 2005–2010 cycles of the National health and nutrition examination survey (NHANES) database. The relationship between OBS and constipation was analyzed by logistic regression, restricted cubic spline. Trend analysis was used to explore whether there is a linear relationship between OBS quartiles and constipation, while interaction analysis was conducted to determine whether other factors influence the relationship between OBS and constipation. Subgroup analysis was used to examine the relationship between the two in different subgroups. The three machine learning algorithms including Xgboost, Randomforest, and AdaBoost was used to analyze the important component of OBS in constipation.

**Results:**

A total of 8,074 participants were involved. We found that there was a negative linear relationship between OBS and constipation. The relationship also existed after adjusting for all possible confounders. The trend test showed that the higher the OBS, the lower the likelihood of developing constipation (*P* for trend<0.05). The interaction analysis showed that marital status and diabetes interact with OBS on constipation. The receiver operating characteristic analysis indicated that OBS had a good prediction efficiency on constipation, especially in participants without diabetes and with the status of married or living with a partner. We also found that the body mass index and magnesium were important OBS components related to constipation.

**Conclusion:**

Oxidation balance score was negatively associated with the occurrence of constipation in adults. Moreover, body mass index and magnesium were important OBS components related to constipation.

## 1 Background

Constipation mainly refers to a group of diseases characterized by clinical manifestations such as difficulty in defecation, small quantity of stool, reduced frequency of defecation, a feeling of incomplete evacuation, and related discomforts (1). According to literature reports, this disease is a common and complex gastrointestinal disorder, affecting approximately 15% of people globally (2). It hurts the quality of life and may lead to serious health complications, so it is worthy of attention. Many factors affect the occurrence of constipation including different diseases, diet, habits and behavior, and medications (3, 4).

Oxidative stress is a harmful condition that occurs in all living systems and is caused by an imbalance between oxidant species and antioxidant defenses (5). Oxidative stress has been implicated in the development of various diseases, including cardiovascular disease (6), osteoporosis (7), neurodegenerative diseases (8), as well as intestinal diseases (9). For instance, research has shown that oxidative stress can lead to the translocation of TDP-43 protein from the nucleus to the cytoplasm and reduce TDP-43 protein levels, resulting in DNA damage and genomic instability. This process activates mitochondria-dependent necrosis in intestinal epithelial cells, thereby inducing the onset of inflammatory bowel disease (10). Other studies have indicated that the total oxidant status and oxidative stress index in the serum of patients with necrotizing enterocolitis (NEC) are elevated and correlate with disease severity, suggesting a relationship between oxidative stress and NEC (11). In addition, inflammation in the body is related to oxidative stress caused by an imbalance between oxidants and antioxidants. Recent studies have shown that medicine can reduce the expression of inflammatory factors in the colon of constipated rats and their serum (12). A higher dietary inflammatory index is associated with a higher risk of constipation (13). *Bifidobacterium animalis* ssp. lactis V9 can alleviate constipation by counteracting inflammatory pathways (1). These studies indicate that constipation is related to inflammation in the body. Therefore, oxidants and antioxidants play an important role in the occurrence of constipation. In general, many studies indicated that low antioxidant intake or high oxidant intake increases the risk of constipation. For example, vitamin D vitamin B1 (14), lactulose (15), and salt (16) have been shown to influence constipation. However, these studies are limited to the relationship between one or a few oxidants or antioxidants and constipation, which cannot assess a relationship between an individual’s overall oxidant exposure level and constipation. Therefore, in this study, we aimed to explore the relationship between the oxidative balance score (OBS) and constipation.

To measure individual pro-oxidant and overall antioxidant exposure, the OBS was invented base on the diet and lifestyle (17). A higher OBS indicates a greater level of antioxidant exposure relative to oxidant exposure. Numerous studies have explored the relationship between OBS and various diseases. For example, a study by Ke et al. (18) demonstrated that higher OBS was associated with a lower risk of kidney stones; those in the highest OBS quartile had a 33% reduced risk of kidney stones compared to those in the lowest quartile. Cho et al. found that higher OBS was significantly associated with a lower risk of non-alcoholic fatty liver disease (19). Zhou and Han (20) indicated that increased OBS was linked to better hearing health in adults, with vitamin B12, total fat, and physical activity in the OBS playing significant roles in hearing loss. Overall, a higher OBS implies a well-balanced diet and healthier lifestyle habits, which enhance antioxidant capacity and reduce oxidative stress in the body, thereby lowering the risk of various diseases, including sarcopenia, urinary incontinence (21), depression (22), and rectal cancer (23). There is limited research on the relationship between constipation and OBS. To contribute to this field, we explored the relationship between OBS and constipation based on National Health and Nutrition Examination Survey (NHANES) public databases and identified OBS components significantly related to constipation.

## 2 Methods

### 2.1 Data sources

All data of this study was collected from the NHANES database. The database is a program of studies designed to assess the health and nutritional status of adults and children in the United States. The survey is unique in that it combines interviews and physical examinations. NHANES is a major program of the National Center for Health Statistics (NCHS). NCHS is part of the Centers for Disease Control and Prevention (CDC) and has the responsibility for producing vital and health statistics for the Nation.

### 2.2 Study design and population

**Figure 1:**
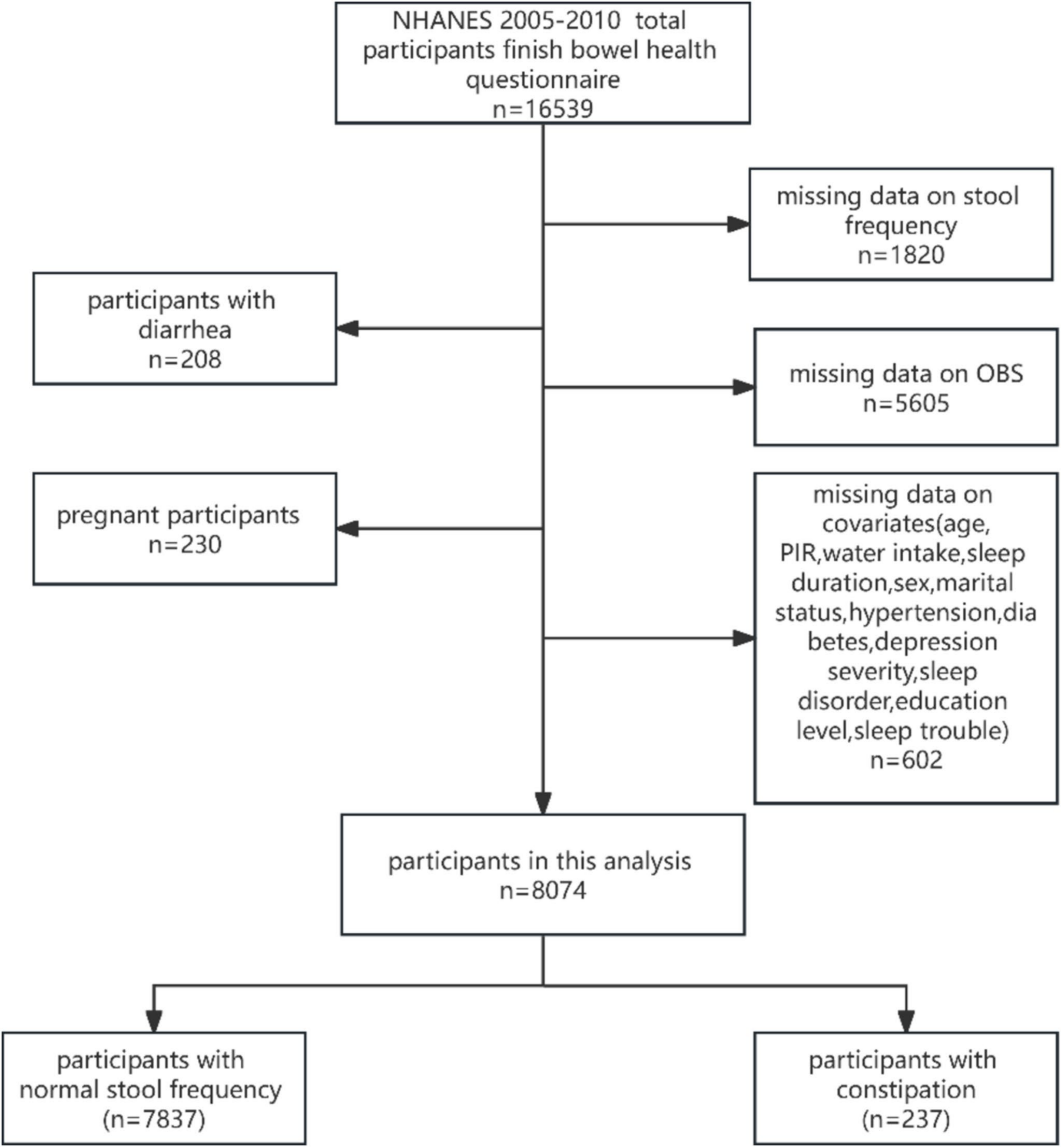
Flow charts of the inclusion and exclusion of participants.

In this study, we collected data from the 2005–2010 year cycle of NHANES because of participants with information on bowel habits in those cycles. The information of 16,539 participants who participated in the bowel health questionnaire was collected initially. Then, those participants were further selected based on inclusion and exclusion criteria. Inclusion criteria: ① participants with information of stool frequency (*n* = 14,719); ② participants with whole OBS factors (*n* = 9,114); ③ participants with complete covariates (*n* = 8,512). Exclusion criteria: ① participants with diarrhea (*n* = 208); ② pregnant participants (*n* = 230). Finally, a total of 8,074 participants were enrolled in this study. Figure 1 shows the screening process flowchart of this study.

### 2.3 Diagnosis of constipation and calculation of OBS

The defecation frequency and stool consistency of the bowel health questionnaire were used to assess the constipation and diarrhea of participants in the NHANES database. However, the defecation frequency was weakly associated with stool consistency (24, 25). Therefore, we diagnosed constipation based on stool frequency that answered the following question: “How often have you had a bowel movement each week over the past 30 days?.” The stool frequency was grouped into three kinds of statuses as follows: stool frequency < 3 was defined as constipation; 3 ≤ stool frequency ≤ 21 as defined as normal; stool frequency > 21 was defined as diarrhea (13, 24).

The OBS contains 16 kinds of dietary intake factors and 4 kinds of lifestyle factors. The dietary intake factors were collected from the first dietary review interview and include vitamin B6, vitamin E, vitamin B12, vitamin C, total folate, calcium, magnesium, zinc, copper, selenium, iron, dietary fiber, carotene, riboflavin, niacin, and total fat. The lifestyle factors included physical activity, body mass index (BMI), alcohol consumption, and smoking. Among them, vitamin E, vitamin B12, vitamin C, total folate, calcium, magnesium, zinc, copper, selenium, dietary fiber, carotene, riboflavin, and niacin were considered antioxidants. BMI, alcohol consumption smoking, total fat, and iron were considered pro-oxidants. OBS was calculated using the method in previously reported literature by Zhang et al. (17).

### 2.4 Covariates

According to the literature, the potential confounders were collected, including age, poverty income ratio (PIR), water intake, sleep duration, sex, marital status, hypertension, diabetes, depression severity, sleep disorder, sleep trouble, and education level. Among them, the marital status was classified into married or living with a partner group and living alone group; the education levels were divided into three groups: above high school, below high school, and high school. The depression severity was divided into five degrees based on the PHQ9 score, including minimal (1–4 score), mild (5–9 score), moderate (10–15score), moderately severe (15–19 score), and severe (20–27 score) (26). The hypertension was diagnosed based on three conditions including the question: “Have doctors ever told you had hypertension?”; taking prescribed medicine to decrease blood pressure; and blood pressure ≥ 140/90 mmHg. Diabetes was diagnosed on the basis of questions: “Have doctors ever told you had diabetes?” or self-reported current use of diabetes medication or insulin. Sleep trouble was diagnosed based on the question: “Ever told doctor had trouble sleeping?.” Sleep disorder was diagnosed according to the question: “Ever told by a doctor have a sleep disorder?”.

### 2.5 Statistical analysis

The statistical analysis used the 1/3 two-year dietary day one sample weight according to the screening and calculation rule of weight from NHANES analysis guidelines via 4.2.2 version R software. The categorical variables were compared between the two groups via a chi-square test, while the continuous variables were compared between the two groups via the *T*-test. A *p*-value less than 0.05 was considered statistically significant. The logistic regression analysis investigated the relationship between OBS score and constipation. The crude model was unadjusted. Model 1 was adjusted for age, PIR, sex, and marital status. Model 2 was adjusted for age, PIR, sex, marital status, and water intake. Model 3 was adjusted for age, PIR, sex, marital status, water intake, hypertension, diabetes, and depression severity. The odder ratios (OR) and 95% confidence interval (CI) were used to estimate the relationship between the OBS and constipation. The trend test between the OBS and constipation was analyzed. In addition, the model was visualized via restricted cubic spline (RCS). The interaction analysis was used to explore the interaction between OBS and confounding factors on constipation, which adjusted for the other confounding factors. The subgroup analysis was performed to screen the population whose OBS was the most suitable for predicting constipation. Moreover, the receiver operating characteristic (ROC) was used to assess the prediction efficiency. Finally, Xgboost, AdaBoost, and Random Forest were performed to evaluate the importance of the OBS components.

## 3 Results

### 3.1 Characteristics of participants in this study

A total of 8,074 participants were divided into two groups: the constipation group and the non-constipation group. There were significant differences between the two groups in terms of age (*p* = 0.005), PIR (*p* < 0.001), water intake (*p* = 0.001), OBS (*p* < 0.001), sex (*p* < 0.001), marital status (*p* = 0.031), hypertension (*p* = 0.019), diabetes (*p* = 0.018), and depression severity (*p* < 0.001). The differences in other characteristics between the two groups were not statistically significant. The comparing results are summarized in Table 1.

**Table 1:** The weighted baseline characteristics of the participants.

Variable	No constipation	Constipation	*P*-value
Age, years	46.190 (0.389)	42.328 (1.314)	0.005
PIR	3.256 (0.044)	2.601 (0.129)	<0.001
Water intake (gm/day)	3201.284 (34.223)	2628.398 (159.307)	0.001
Sleep duration, h	6.895 (0.023)	6.945 (0.116)	0.668
OBS	21.959 (0.184)	19.143 (0.641)	<0.001
Sex, %			<0.001
Female	3,654 (48.588)	186 (86.097)	
Male	4,183 (51.412)	51 (13.903)	
Marital status, %			0.031
Living alone	2,849 (33.919)	107 (42.688)	
Married or living with partner	4,988 (66.081)	130 (57.312)	
Education level			0.154
Above high school	4,284 (63.179)	117 (56.054)	
Below high school	1741 (13.906)	52 (16.111)	
High school	1812 (22.914)	68 (27.835)	
Hypertension, %			0.019
No	4,800 (65.635)	164 (75.689)	
Yes	3,037 (34.365)	73 (24.311)	
Diabetes, %			0.018
No	6,229 (84.121)	205 (90.468)	
Yes	1,608 (15.879)	32 (9.532)	
Depression severity, %			<0.001
Minimal	6,226 (80.476)	147 (63.894)	
Mild	1,099 (13.916)	45 (17.614)	
Moderate	344 (3.699)	30 (13.068)	
Moderately severe	130 (1.559)	14 (4.665)	
Severe	38 (0.349)	1 (0.760)	
Sleep trouble, %			0.132
No	6,041 (75.868)	166 (70.133)	
Yes	1796 (24.132)	71 (29.867)	
Sleep disorder, %			0.617
No	7,303 (93.036)	221 (91.729)	
Yes	534 (6.964)	16 (8.271)	

PIR, poverty income ratio; OBS, oxidative balance score.

### 3.2 Relationship between OBS and constipation

**Figure 2:**
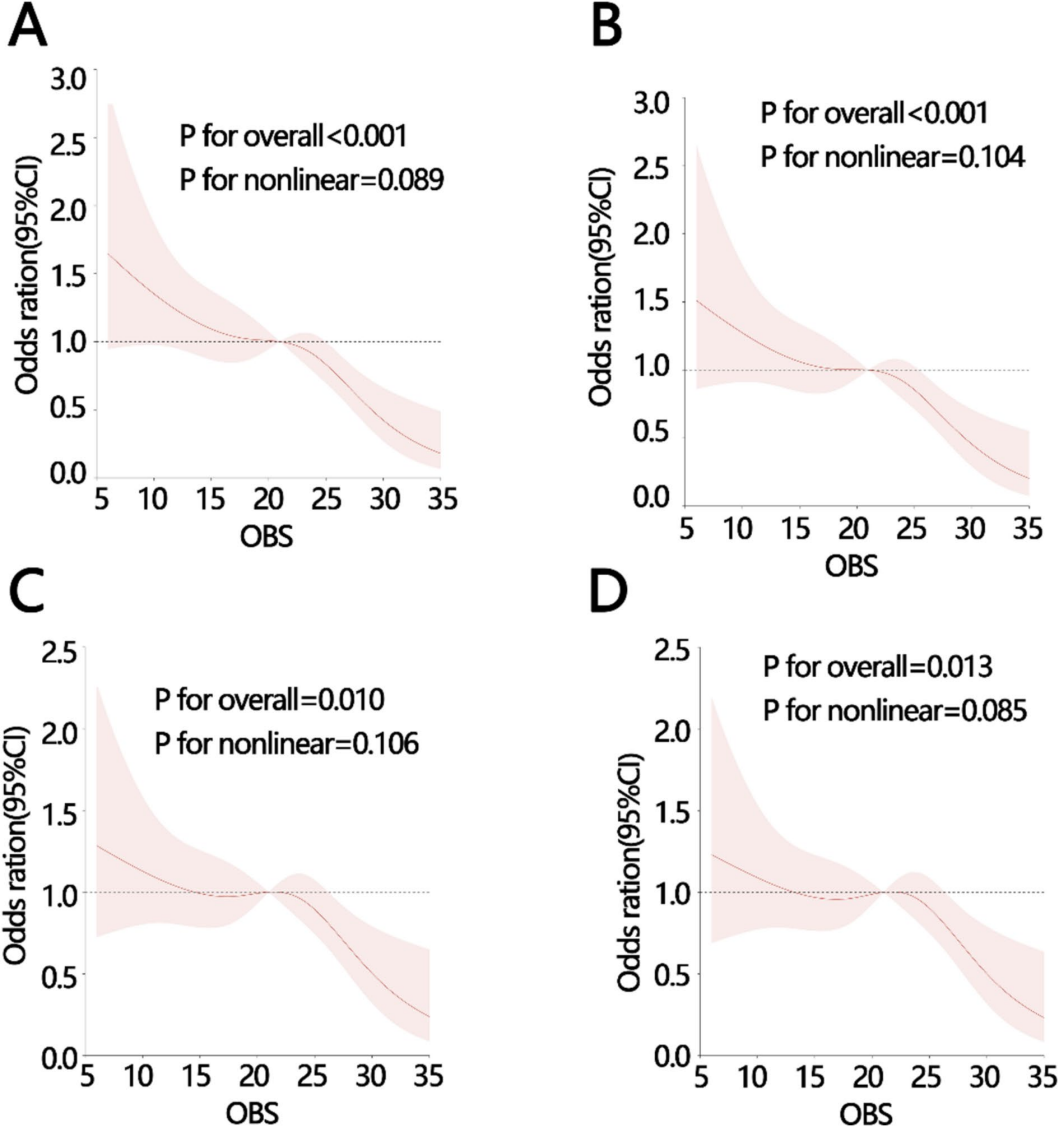
The RCS analysis between OBS and constipation with different adjustments (A) crude model: without adjustment, (B) model 1: adjusting for age, PIR, sex, and marital status, (C) model 2: adjusting for, age, PIR, sex, marital status, water intake, (D) model 3: adjusting for, age, PIR, sex, marital status, water intake, Hypertension, diabetes, depression severity. PIR, poverty income ratio; OBS, oxidative balance score.

The weighted logistic regression analysis was used to investigate the relationship between the OBS and constipation. As shown in Table 2, the results indicated that OBS was an obstruction factor for the occurrence of constipation when the OBS served as a continuous variable (OR 95%CI: −0.002[−0.002, −0.001], *p* < 0.001). The results also existed even adjusting for confounding factors in model1 (OR 95%CI: −0.001[−0.002, −0.001], *p* < 0.001), model2 (OR 95%CI: −0.001[−0.002, 0.000], *p* = 0.004), model3 (OR 95%CI -0.001[−0.002, 0.000], *p* = 0.006). The quartiles of OBS and constipation were also analyzed using trend analysis to further explore the relationship between OBS and constipation. The result showed that the higher the OBS, the lower the likelihood of occurrence of constipation, and the result remained significant in model 1, model 2, and model 3, respectively (all *P* for trend <0.05, Table 2). Using model 1 as an example, for every 10-unit increase in OBS, the probability of constipation decreases by 20%. Taking Q1 as a reference, individuals with OBS levels at Q2 have a 7% lower probability of experiencing constipation, those at Q3 have a 17% lower probability, and individuals at OBS Q4 level have a 28% lower probability of experiencing constipation. Those models were adjusted for the potential confounding factors. Therefore, we speculated that there may be a linear relationship between OBS and constipation, and used RCS to verify it. The RCS results showed a linear relationship between them, even after adjusting for different confounding factors (all *P* for overall < 0.05; all *P* for non-linear > 0.05; Figures 2A–D).

**Table 2:** Logistic regression analysis investigating the relationship between OBS score/quartiles of OBS and constipation.

Variable	Crude model		Model 1		Model 2		Model 3	
Character	95%CI	*P*-value	95%CI	*P*-value	95%CI	*P*-value	95%CI	*P*-value
OBS (continuous)	−0.002(−0.002, −0.001)	<0.001	−0.001(−0.002, −0.001)	<0.001	−0.001(−0.002, 0.000)	0.004	−0.001(−0.002, 0.000)	0.006
OBS (categories)								
Q1	Ref		Ref		Ref		Ref	
Q2	−0.007(−0.021, 0.007)	0.333	−0.006(−0.020, 0.008)	0.371	−0.005(−0.019, 0.009)	0.463	−0.003(−0.017, 0.011)	0.672
Q3	−0.017(−0.032, −0.002)	0.023	−0.014(−0.029, 0.000)	0.058	−0.013(−0.028, 0.003)	0.108	−0.011(−0.026, 0.004)	0.158
Q4	−0.028(−0.040, −0.016)	<0.001	−0.026(−0.038, −0.013)	<0.001	−0.023(−0.037, −0.009)	0.002	−0.021(−0.034, −0.007)	0.004
*p* for tend		<0.001		<0.001		0.002		0.004

Crude model: without adjustment.

Model 1: adjusting for age, PIR, sex, and marital status.

Model 2: adjusting for age, PIR, sex, marital status, and water intake.

Model 3: adjusting for age, PIR, sex, marital status, water intake, Hypertension, diabetes, and depression severity.

PIR, poverty income ratio; OBS, oxidative balance score.

### 3.3 Predictive value and important component of OBS in constipation

**Figure 3:**
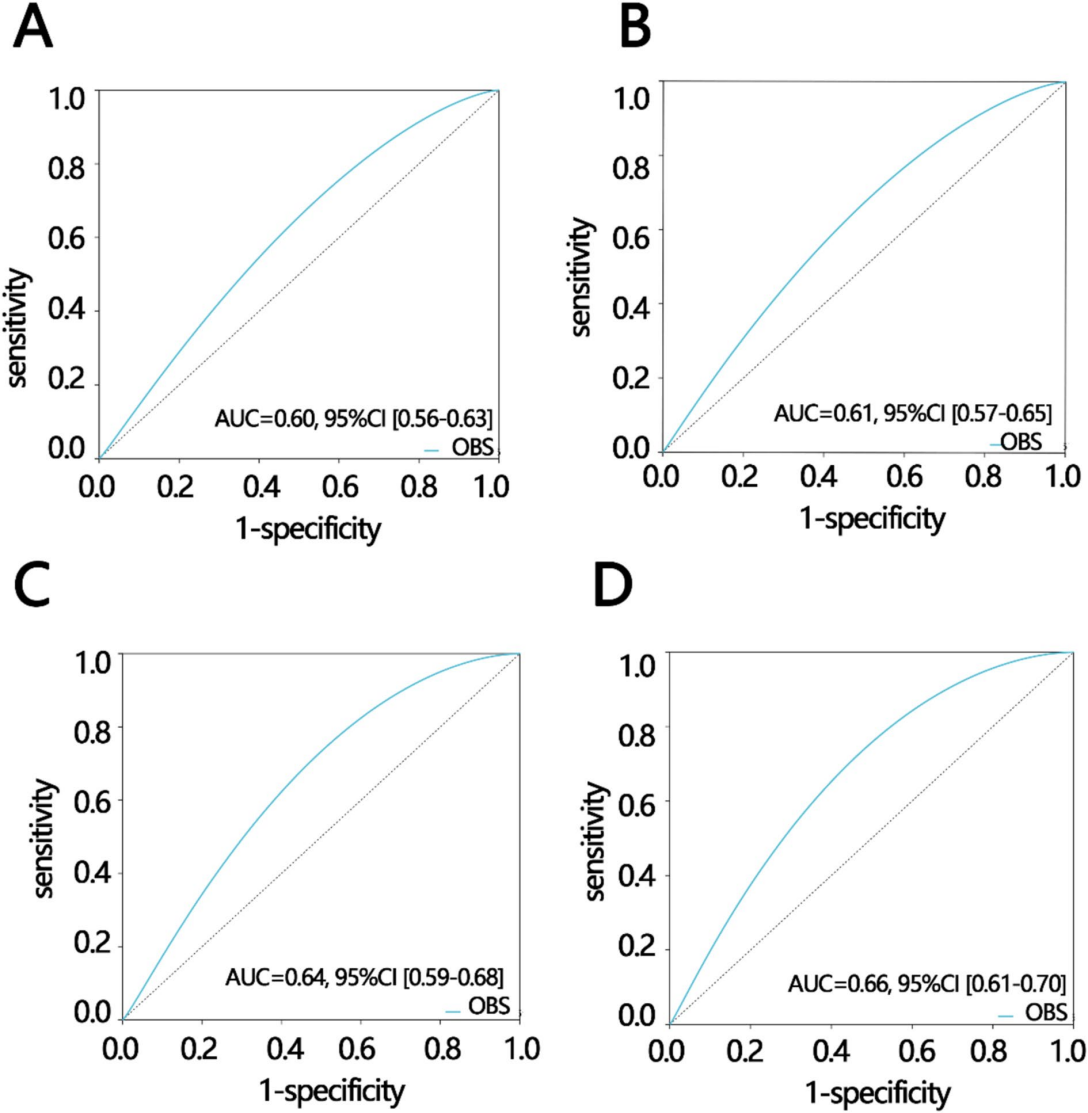
Receiver operating characteristic analysis in different participant groups including (A) all participants, (B) participants without diabetes, (C) participants with status of married or living with partner, (D) participants without diabetes and with the status of married or living with partner. AUC, areas under curve; CI, confidence interval.

To explore the predictive value of OBS in constipation, we initially used interaction analysis to obtain confounding factors that interact with OBS on constipation. Next, weighted logistic regression analysis was performed based on these factors to obtain the important subgroups. The interaction analysis showed that marital status and diabetes interact with OBS on constipation after adjusting for confounding factors other than itself (*P* for interaction < 0.05, Table 3). There was no display of confounding factors without significant differences in the interaction including age, PIR, sex, water intake, Hypertension, and depression severity. Finally, we obtained two important subgroups including participants who were married or living with a partner (OR 95%, CI 0.912[0.886, 0.939], *p* < 0.001), and participants without diabetes (OR 95%, CI 0.934[0.909, 0.959], *p* < 0.001) (Table 3). In addition, we used the ROC analyses to explore the value of OBS in predicting constipation in all populations and important subgroup populations. The results of ROC indicated that OBS had the best prediction efficiency in participants who were married or living with a partner and did not have diabetes, compared with the other three groups including all participants group, married or living with a partner participants group, and without diabetes participants group (AUC: all participants: 0.60; participant without diabetes: 0.61; participants with status of married or living with partner: 0.64; participants without diabetes and with status of married or living with partner: 0.66, Figures 3A–D).

**Table 3:** Association between OBS and constipation stratified by relevant variables.

Character	95% CI	*P*-value	*P* for interaction
Marital status			<0.001
Married or living with partner	0.912 (0.886,0.939)	<0.001	
Living alone	0.986 (0.954,1.019)	0.395	
Diabetes			0.041
No	0.934 (0.909,0.959)	<0.001	
Yes	1.007 (0.941,1.078)	0.837	

Marital status subgroup: adjusting for age, PIR, sex, water intake, hypertension, diabetes, depression severity.

Diabetes subgroup: adjusting for age, sex, PIR, water intake, hypertension, diabetes, depression severity.

PIR, poverty income ratio; OBS, oxidative balance score.

**Figure 4:**
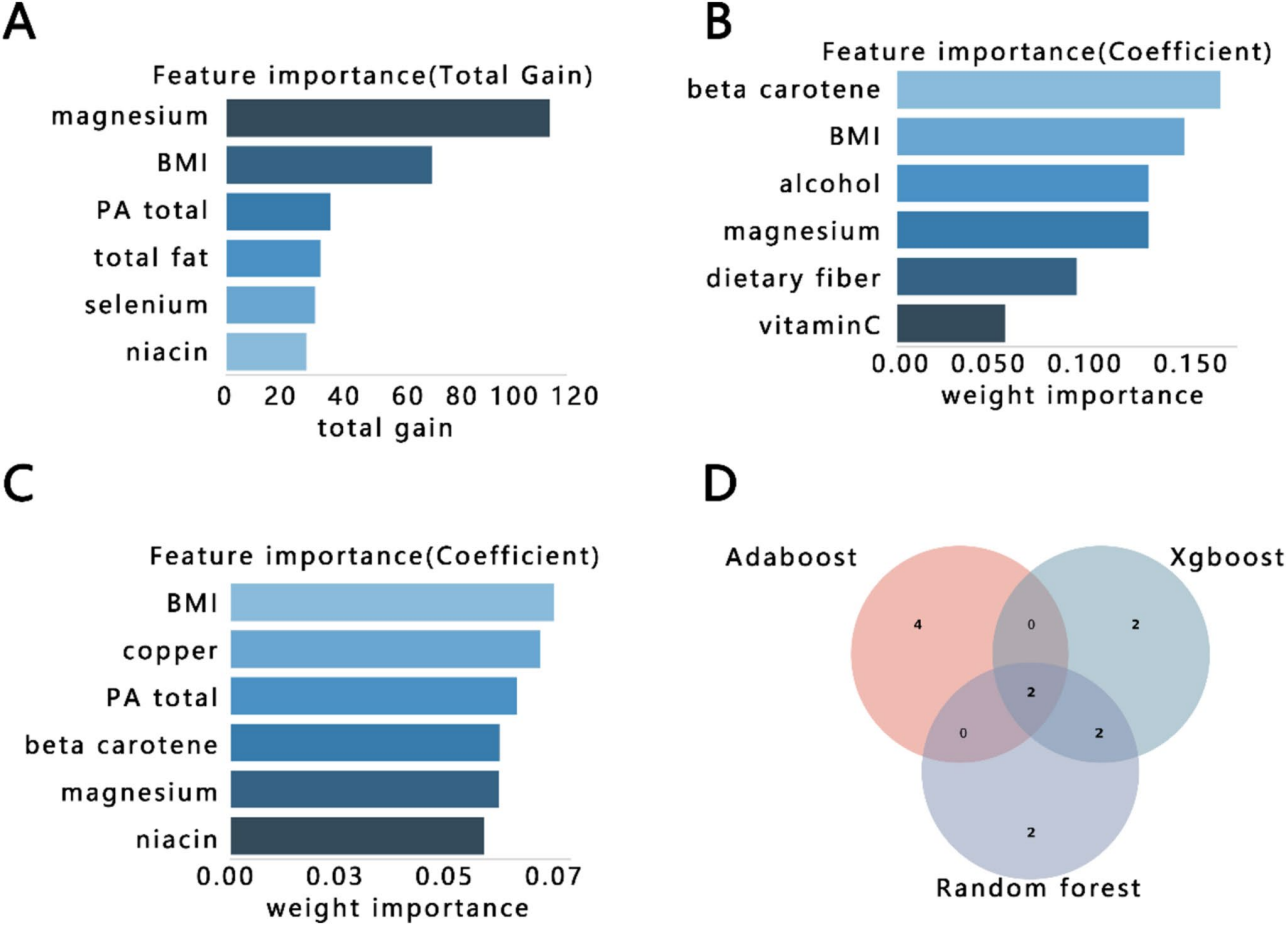
The importance ranking bar chart of the top six OBS factors via three methods. (A) Xgboost, (B) AdaBoost, (C) Random forest, (D) Venn plot of three kinds of rank variables. BMI, body mass index; PA, physical activity.

The above results showed that OBS was an important independent impact factor in constipation. Finally, we further explore its important component in constipation via three importance ranking methods as follows: Xgboost, AdaBoost, and Random Forest. The first six important components are shown in Figures 4A–C. Finally, we screened the two uniform components, BMI and magnesium, via Venn plot (Figure 4D).

## 4 Discussion

Constipation is a common and complex gastrointestinal disorder that affects patients’ quality of life. In this study, we aimed to explore the relationship between OBS and constipation based on the NHANES database. We found that the higher the OBS, the lower the probability of constipation. There was a negative linear relationship between the OBS and constipation. In addition, the BMI and magnesium contributed more to the relationship between OBS and constipation. Those results indicated that an antioxidant diet and lifestyle could inhibit the occurrence of constipation.

In this study, it was found that a higher OBS correlates with a lower probability of constipation. This is because a higher OBS indicates greater antioxidant exposure compared to oxidant exposure, implying higher levels of antioxidants in the body, reduced oxidative stress, and lower reactive oxygen species (ROS) levels (27). Numerous studies have demonstrated that antioxidants and pro-oxidants can directly stimulate the gastrointestinal tract because they can directly influence the levels of ROS (28). ROS levels are closely associated with gut microbiota homeostasis, intestinal inflammation, and gut permeability (29, 30), and are key factors in gastrointestinal diseases (28). Nicotinamide Adenine Dinucleotide Phosphate (NADPH) oxidase is a major producer of reactive oxygen species and plays a crucial role in antimicrobial host defense and oxidative stress in the colon. Studies have shown that NADPH oxidase can cause dysbiosis of the gut microbiota, leading to constipation (31). The occurrence of constipation was directly related to intestinal motility, with gut microbiota being an important factor associated with intestinal peristalsis (32). Furthermore, a higher OBS reduces oxidative stress, resulting in lower ROS levels, which may inhibit the NF-κB pathway, subsequently reducing the production of IL-6 and other inflammatory factors. This helps maintain a stable gut microbiota environment and decreases the likelihood of constipation (33). Reduced oxidative stress may also favor the development of beneficial gut bacteria, which can regulate bile acid synthesis. Bile acids can activate the Takeda G-protein-coupled – receptor 5 on enterochromaffin cells, leading to the release of serotonin and calcitonin gene-related peptide, thereby stimulating the enteric nervous system and altering gut motility (34, 35). Additionally, beneficial bacteria can influence fatty acid synthesis, promoting the synthesis of unsaturated fatty acids, which can accelerate the recovery of intestinal motility, thereby inhibiting constipation (36).

In addition, our study also found that BMI and magnesium play significant roles in the negative correlation between OBS and constipation. It is well-known that magnesium ions have a long history of being used as a treatment for constipation, including compounds like magnesium oxide, magnesium sulfate, and magnesium nitrate. The mechanism by which magnesium treats constipation involves the conversion of magnesium ions into magnesium carbonate and magnesium bicarbonate within the body, thereby increasing the osmotic pressure within the intestinal lumen. This process draws water into the intestinal lumen, increasing the water content and volume of feces, which stimulates the intestinal wall and promotes peristalsis (37, 38). Numerous studies have shown a close relationship between BMI and constipation. For instance, research by Bouchoucha et al. (39) indicated a significant negative correlation between BMI and colonic transit time. Constipated patients in the overweight group had shorter rectosigmoid and overall colonic transit times compared to those in the normal BMI group. In other words, a higher BMI is associated with an increased risk of constipation (39). One cross-sectional study based on NHANES by Xiang et al. (40) suggested that lowering BMI reduces constipation risk. These findings underscore the importance of maintaining healthy dietary habits and a balanced lifestyle to prevent constipation.

Based on our findings and the review by Bellini et al. (41) on nutritional therapy for chronic constipation, we propose the following recommendations to help prevent the onset of constipation: (1) Consume more antioxidant-rich foods: Include foods like kiwifruit, psyllium, plums, figs, flaxseeds, and similar items that have strong antioxidant properties. (2) Increase mineral water intake: magnesium compounds in mineral water exert an osmotic effect, which helps to soften stool. (3) Increase dietary fiber intake: Include more whole grains, vegetables, fruits, and legumes in your diet. Fiber can be fermented by gut microbiota, producing gas and short-chain fatty acids that stimulate bowel movements. (4) Engage in regular physical activity: Adopt a more active and healthy lifestyle, as exercise promotes better bowel function.

### 4.1 Strengths and limitations

The study has some strengths. First, this study had a large sample size based on the NHANES. Second, we strictly abide by the data analysis principles of the database to obtain more reliable conclusions. Third, we screened the important OBS component in constipation. However, there were also some limitations. First, the causality between the OBS and constipation was still unclear due to the cross-sectional study. Second, we did not collect the inflammation index to verify their connection. Third, OBS does not cover all the factors of antioxidant and pro-oxidative exposure, and can only measure individual oxidation balance levels to some degree. Fourth, the diagnosis of constipation via stool frequency may is not accurate. Fifth, the potential bias introduced by self-reported bowel movements and dietary intakes in NHANES data, which might affect the accuracy of the constipation diagnosis and OBS calculation. We found that our findings contrast with the only other study that explored the relationship between OBS and constipation based on the NHANES database, which showed a positive correlation between constipation and OBS (42). In the study by Hu et al. (42), constipation and diarrhea were defined based on the Bristol stool scale classification, whereas in our study, they were defined based on stool frequency, resulting in differences in the number of participants classified as having diarrhea, constipation, or normal stool patterns. Additionally, there were differences in the confounding factors adjusted for: in our study, we adjusted for age, PIR, sex, marital status, water intake, hypertension, diabetes, and depression severity, whereas their study adjusted for age, sex, race, education level, marital status, PIR, milk intake, liquid intake, carbohydrate intake, sugar intake, protein intake, caffeine intake, depression, and the number of comorbidities. These differences in the definition criteria and confounding factors may have led to the divergent study conclusions. Methodologically, each of the two studies has its own strengths and limitations, and it is inappropriate to judge one as simply correct or incorrect. Therefore, the relationship between them needs to be further explored.

## 5 Conclusion

This study released the negative linear relationship between OBS and constipation, which provided a new reference for understanding the relationship between oxidant exposure and constipation. OBS had better predictive value for predicting constipation in participants without diabetes and with the status of married or living with a partner. In addition, BMI and magnesium were important OBS components in constipation.

## Data Availability

The datasets presented in this study can be found in online repositories. The names of the repository/repositories and accession number(s) can be found: the datasets analyzed in this study are available from the NHANES databases (available from https://www.cdc.gov/nchs/nhanes/participant.htm).
